# Amorphization of Thiamine Mononitrate: A Study of Crystallization Inhibition and Chemical Stability of Thiamine in Thiamine Mononitrate Amorphous Solid Dispersions

**DOI:** 10.3390/ijms21249370

**Published:** 2020-12-09

**Authors:** Seda Arioglu-Tuncil, Adrienne L. Voelker, Lynne S. Taylor, Lisa J. Mauer

**Affiliations:** 1 Department of Food Science, Purdue University, 745 Agriculture Mall Drive, West Lafayette, IN 47907, USA; starioglu@beu.edu.tr (S.A.-T.); avoelke@purdue.edu (A.L.V.); 2 Department of Industrial and Physical Pharmacy, Purdue University, 575 Stadium Mall Drive, West Lafayette, IN 47907, USA; lstaylor@purdue.edu

**Keywords:** vitamin B_1_, thiamine, thiamine mononitrate, thiamine degradation, amorphous solid dispersion, physical stability, chemical stability, amorphous stability

## Abstract

This study investigated thiamine degradation in thiamine mononitrate (TMN):polymer solid dispersions, accounting for the physical state of the vitamin and the recrystallization tendency of TMN in these dispersions. Results were compared with those from solid dispersions containing a different salt form of thiamine (thiamine chloride hydrochloride (TClHCl)). TMN:polymer dispersions were prepared by lyophilizing solutions containing TMN and amorphous polymers (pectin and PVP (polyvinylpyrrolidone)). Samples were stored in controlled temperature and relative humidity (RH) environments for eight weeks and monitored periodically by X-ray diffraction and high performance liquid chromatography (HPLC). Moisture sorption, glass transition temperature (T_g_), intermolecular interactions, and pH were also determined. Similar to the TClHCl:polymer dispersions, thiamine was more chemically labile in the amorphous state than the crystalline state, when present in lower proportions in amorphous TMN:polymer dispersions despite increasing T_g_ values, when environmental storage conditions exceeded the T_g_ of the dispersion, and when co-formulated with PVP compared to pectin. When thiamine remained as an amorphous solid, chemical stability of thiamine did not differ as a function of counterion present (TMN vs. TClHCl). However, storage at 75% RH led to hydration of thiamine:PVP dispersions, and the resulting pH of the solutions as a function of thiamine salt form led to a higher chemical stability in the acidic TClHCl samples than in the neutral TMN samples.

## 1. Introduction

Thiamine is one of the most unstable vitamins: it is highly affected by heat, light, alkaline pH, the presence of some food ingredients, radiation, inorganic bases, and processing treatments [1,2,3]. The susceptibility of thiamine to these conditions makes its delivery in processed, shelf-stable foods and dietary supplements challenging. When thiamine degrades, off-flavors develop due to formation of sulfur-containing degradation products [2,4,5,6], thereby affecting not only vitamin label claims but also product acceptability as degradation proceeds. Therefore, understanding factors that affect the stability of thiamine in foods and dietary supplements is important for shelf-life, nutritional labeling, and food quality considerations.

Two synthetic crystalline salt forms of thiamine, thiamine mononitrate (TMN) and thiamine chloride hydrochloride (TClHCl), have been extensively used for enrichment and fortification purposes in foods as well as in dietary supplements. These salt forms of thiamine possess different physicochemical features. For example, TMN is less soluble and less hygroscopic than TClHCl; thus, TMN is commonly used to enrich dry food products, including flour, and in vitamin tablets and dietary supplements [7]. Although both TMN and TClHCl are distributed as crystalline ingredients, some processing methods and/or interactions with other food ingredients, such as polymers, may lead to the presence of thiamine in the amorphous state in many low or intermediate moisture products [8]. A study of TClHCl in solid dispersions found that polymers (such as pectin (PEC)) that could form the most extensive hydrogen bonding and/or ionic interactions with TClHCl were the most effective crystallization inhibitors, extending the physical stability of TClHCl in the amorphous state better than polymers (such as polyvinylpyrrolidone (PVP)) with fewer intermolecular interactions with the vitamin form [8]. Amorphous forms of ingredients tend to be more chemically labile than their crystalline counterparts [9], which was found to be true for the thiamine in the TClHCl solid dispersions; however, differences in the chemical stability of thiamine in the amorphous state in the presence of different polymers were also found [10]. In the amorphous TClHCl solid dispersions, thiamine was more chemically stable in the presence of pectin compared to PVP, which can be attributed to the increased intermolecular interactions between pectin and thiamine and the more acidic nature of pectin [10].

The interesting insights into the physical and chemical stability of thiamine in amorphous TClHCl solid dispersions developed in previous work [8,10] are extended here to the ingredient form of thiamine (TMN) that is more often used in low and intermediate moisture foods and dietary supplements, wherein crystallization and degradation in different solid states are highly relevant. Although TMN is potentially found in its amorphous form in food and dietary supplement products, there has been no study conducted to investigate its physical and chemical stability in the amorphous form. It was anticipated that co-formulation of TMN with polymers would result in successful amorphization of the vitamin due to its interactions with polymeric ingredients, similar to what was found in the TClHCl:polymer dispersions [8]. The effects of counterion and pH differences on the stability of thiamine in the TClHCl versus TMN solid dispersions were of interest. Therefore, the objectives of this study were: (1) to investigate physical and chemical stability of amorphous TMN in solid dispersions made using PVP and pectin (which were selected based on their T_g_, hydrogen bonding ability, molecular structures, and hygroscopicity); (2) to compare thiamine degradation in the amorphous and crystalline state; (3) to determine the factors causing chemical instability of thiamine in amorphous form; and (4) to compare thiamine stability in polymer dispersions containing TMN to previous reports of those containing TClHCl [8,10].

## 2. Results and Discussion

### 2.1. Physical Stability of Amorphous Thiamine Mononitrate

**Figure 1:**
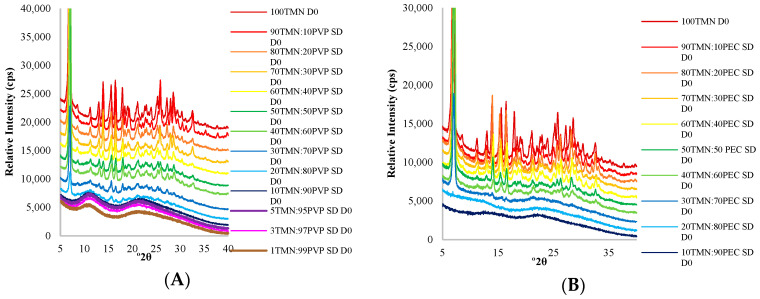
X-ray powder diffraction patterns of (**A**) TMN:PVP solid dispersions (SD) prepared with varying ratios of thiamine mononitrate (TMN) to polyvinylpyrrolidone (PVP) immediately following lyophilization; and (**B**) thiamine mononitrate:pectin (TMN:PEC) solid dispersions (SD) prepared with varying ratios of TMN to PEC immediately following lyophilization. The diffractograms are presented from top to bottom in decreasing order of TMN concentration.

**Figure 2:**
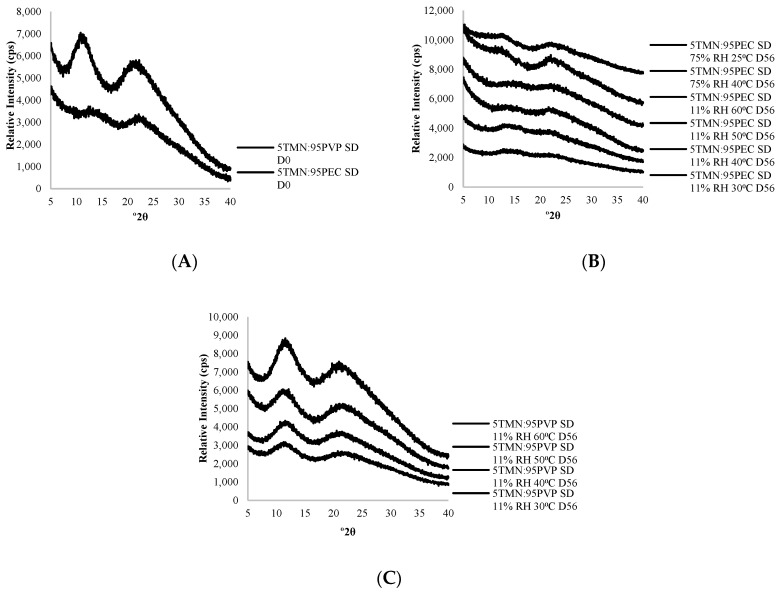
X-ray powder diffraction patterns of: (**A**) 5TMN:95PEC and 5TMN:95PVP solid dispersions (SD) immediately following lyophilization; (**B**) 5TMN:95PEC solid dispersions (SD) stored at 11% relative humidity (RH), 30–60 °C and 75% RH, 25 or 40 °C on day 56; and (**C**) 5TMN:95PVP solid dispersions (SD) stored at 11% RH and 30–60 °C on day 56.

TMN was successfully amorphized in the presence of both PVP and PEC (Figure 1). To form fully amorphous dispersions, at least 90% PVP or 80% PEC was necessary to initially stabilize TMN in the amorphous state in the solid dispersions (Figure 2). A higher ratio of polymer was needed to amorphize TMN compared to TClHCl. Only 60% PVP or 40% PEC was required to fully amorphize TClHCl [8], indicating that TMN may have a higher crystallization tendency than TClHCl. For both thiamine salt forms, more PVP was needed to form amorphous dispersions of the vitamin than PEC, indicating that different traits between the polymers, in addition to the salt form of thiamine, influenced the formation of amorphous solid dispersions.

No evidence of TMN crystallization was found in PXRD patterns of TMN:polymer dispersions that were initially amorphous (TMN:PVP containing ≤ 10% TMN and TMN:PEC containing ≤20% TMN) in samples stored at 11% RH over the 8 week storage study, regardless of storage temperature. PXRD patterns of amorphous 5TMN:95polymer dispersions before and after storage are shown in Figure 2. Increasing the storage RH to 75% RH did not lead to TMN crystallization in the 5TMN:95PEC dispersions (Figure 2B). 

At the 75% RH storage conditions, the 5TMN:95PVP dispersions liquefied, and thus no PXRD patterns were collected. In comparison, TClHCl:polymer dispersions that were initially amorphous remained so during storage at low RHs (e.g., ≤32% RH for 50TClHCl:50PEC dispersions), but increasing the storage RH resulted in TClHCl crystallization in many of the polymer dispersions [8].

### 2.2. Effect of Polymer Type on Amorphization and Physical Stability of TMN Dispersions

Hydrogen bonding between a low molecular weight compound and a polymer has often been cited as an important stabilizing factor for both amorphization of the small molecule and physical stability of the amorphous solid dispersions against recrystallization, in which hydrogen bonding propensity is dependent on hydrogen bond donor (HBD) and acceptor (HBA) groups on the two materials [8,11,12,13,14,15]. In FTIR spectra, peak shifts to lower wavenumbers in spectra of vitamin:polymer dispersions compared to spectra of pure polymers indicate more extensive and/or stronger intermolecular interactions. Additionally, the presence or absence of distinct peaks in the OH/NH stretching region (3600–3000 cm^−1^) of TClHCl:polymer spectra corresponded to the PXRD data such that the FTIR spectra were also indicative of presence/absence of the crystalline salt form of thiamine in the dispersion [8]. In our previous study of amorphous TClHCl polymer dispersions, FTIR spectra indicated substantial hydrogen bonding between TClHCl and PEC, attributed to the HBAs on thiamine and HBDs on PEC, but there was little-to-no hydrogen bonding observed between TClHCl and PVP, attributed to the lack of HBDs on PVP [8]. This difference in hydrogen bonding corresponded to differences in physical stability: more PVP was needed to inhibit TMN crystallization than PEC [8].

**Figure 3:**
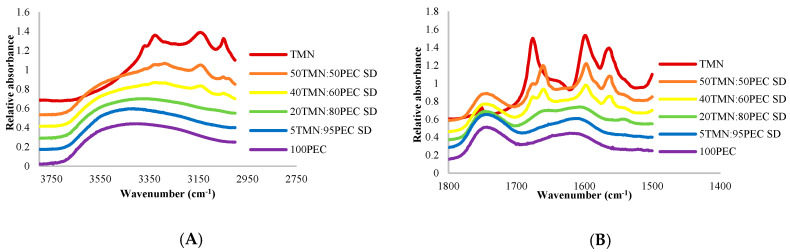

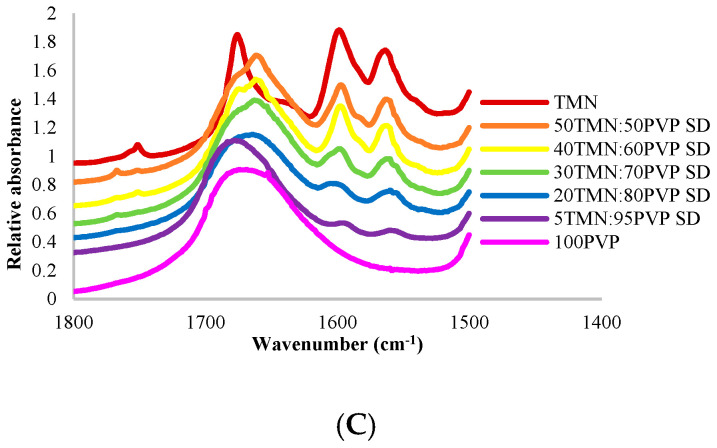
FTIR spectra of the (**A**) hydroxyl region of TMN:PEC solid dispersions, (**B**) carbonyl region of TMN:PEC solid dispersions, and (**C**) carbonyl region of TMN:PVP solid dispersions. The spectra are presented from top to bottom in decreasing order of TMN concentration.

A similar analytical approach was used for the TMN:polymer dispersions. Spectra of crystalline TMN exhibited three distinct peaks in the OH/NH spectral region, located at 3329, 3141 and 3047 cm^−1^ (Figure 3A), and several other peaks were found in the double bond region of the TMN spectra (1800–1500 cm^−1^) (Figure 3B). In TMN:PEC dispersions in which TMN remained crystalline according to PXRD analyses (those containing <80% PEC), the distinct crystalline TMN peaks in both the OH/NH and double bond regions remained in the FTIR spectra (Figure 3A,B). Upon successful amorphization of TMN in the polymer dispersions according to PXRD analyses (in TMN:PEC dispersions containing ≥ 80% PEC), these distinct peaks disappeared, and instead broad peaks were formed in both regions of the FTIR spectra. Thus, as for the TClHCl:polymer dispersions [8], PXRD and FTIR analyses identified the same TMN:polymer ratios needed to prevent TMN crystallization in the initial TMN:polymer lyophiles.

Differences were found in peak shifts in FTIR spectra of varying ratios and types of polymers in the TMN:polymer dispersions between the polymer types. The maximum peak in the hydroxyl region of spectra of pure PEC polymer occurred at 3419 cm^−1^, and this peak shifted to 3363 cm^−1^ in the spectra of amorphous dispersions containing 20TMN:80PEC (Figure 3A). A smaller peak shift was found in the double bond region, from 1622 cm^−1^ in the spectra of PEC alone to 1606 and 1610 cm^−1^ in the amorphous 20TMN:80PEC and 5TMN:95PEC dispersions, respectively (Figure 3B). The large peak shift in the hydroxyl region of the spectra indicates hydrogen bonding occurred between TMN and PEC in amorphous dispersions. Unlike PEC, however, the only functional group of PVP that can participate in hydrogen bonding is the amide carbonyl group (HBA), which absorbs at 1669 cm^−1^ in the double bond region of the FTIR spectra (Figure 3C). However, in amorphous solid dispersions with TMN, this peak either shifted to a higher wavenumber, indicating greater interaction between PVP molecules (less interaction between TMN and PVP), or remained in the same location, indicating that there were limited-to-no hydrogen bonding and/or intermolecular interactions between PVP and TMN, similar to what was seen in spectra of TClHCl:PVP dispersions [8]. The relatively stronger and/or more extensive hydrogen bonding/intermolecular interactions between TMN and PEC compared to TMN and PVP was likely the reason that less PEC was required to amorphize TMN than PVP.

Comparing the peak shifts in spectra of TMN:PEC dispersions to those previously reported for TClHCl:PEC dispersions [8] provides evidence as to why more PEC was needed to amorphize TMN than TClHCl. A larger peak shift (148 cm^−1^) was found in the OH/NH spectral region of the TClHCl:PEC dispersions than the 56 cm^−1^ shift in the same spectral region of the TMN:PEC dispersions, indicating that there were more extensive and/or stronger intermolecular hydrogen bonding interactions between the TClHCl and PEC than between the TMN and PEC. The fact that there is an additional hydrogen bond donor in the structure of TClHCl compared to TMN was likely the contributing factor to this difference. Because TMN had one less hydrogen bond donor than TClHCl, more polymer was needed to stabilize the TMN in the amorphous state than TClHCl.

### 2.3. Effect of Physical State on Chemical Stability of Thiamine

**Figure 4:**
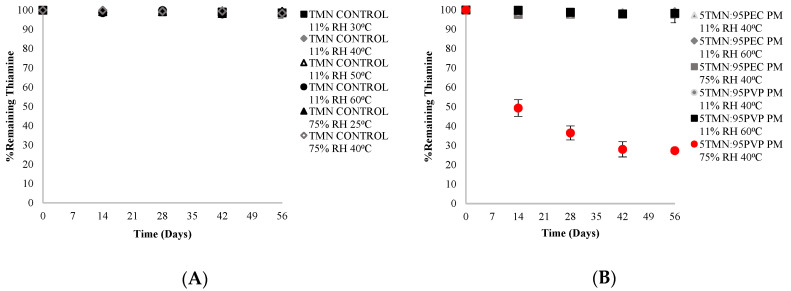
Chemical stability of thiamine (**A**) in pure crystalline TMN form, and (**B**) in 5TMN:95PEC and 5TMN:95PVP physical mixtures (PM).

**Figure 5:**
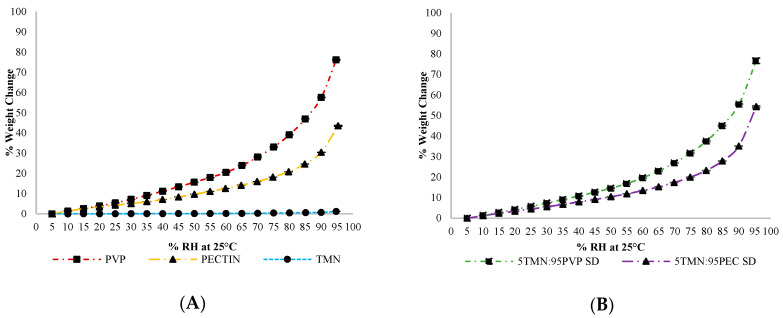
Moisture sorption profiles of (**A**) pure PVP, PEC, and TMN; and (**B**) solid dispersions of 5TMN:95PVP and 5TMN:95PEC following lyophilization at 25 °C.

The physical state (amorphous vs. crystalline) of a compound is known to influence its chemical stability. Amorphous materials, including thiamine, are generally less chemically stable than their crystalline counterparts [10,16]. In the current study, the chemical stability of thiamine in the crystalline state was measured over an 8-week period of storage in different environments (at 11% RH and 30–60 °C and 75% RH and 25–40 °C) in three sample types: (1) pure crystalline TMN (control); (2) physical mixtures containing 5% crystalline TMN and 95% PEC; and (3) physical mixtures containing 5% crystalline TMN and 95% PVP. Regardless of the storage RH and temperature, no significant (*p* < 0.05) thiamine degradation was found in the neat crystalline TMN control samples over the 8 weeks (Figure 4A). Similarly, there was no significant (*p* < 0.05) thiamine degradation in TMN physical blends with PEC or PVP throughout the study, with one exception (Figure 4B). In TMN:PVP physical blends stored at 75% RH and 40 °C, more than 70% thiamine loss was found over the 8-week period. This was presumably due to the hygroscopic nature of PVP, especially at 75% RH. Moisture sorption studies indicated that moisture sorption of both the TMN:polymer dispersions and the physical mixtures was governed by the hygroscopicity of the polymers rather than TMN at these ingredient ratios and storage conditions. Neat PVP absorbed 15% more moisture than PEC at 75% RH (33% and 18% w/w in PVP and PEC, respectively) (Figure 5A), and this increased amount of moisture sorption by PVP in the physical blends led to at least partial dissolution of the crystalline TMN. Since thiamine is less stable in solution than in the solid state [5], the partial dissolution of crystalline TMN in the physical mixture led to extensive degradation at this high RH storage condition. However, TMN that remained crystalline was shown to be chemically stable in the current study, with no significant degradation after 8 weeks in any storage condition (Figure 4). Differences in hygroscopicity of the polymers led to similar differences in hygroscopicity of the solid dispersions, wherein dispersions containing PVP absorbed more water than those containing PEC: the 5TMN:95PVP dispersions absorbed 12% more moisture than the 5:TMN:95PEC dispersions at 75% RH (32% and 20% w/w in 5TMN:95PVP and 5:TMN:95PEC, respectively) (Figure 5B). The increased moisture sorption by the dispersions containing PVP led to their hydration when stored at 75% RH, consistent with the dissolution of crystalline TMN in a physical mixture with PVP stored at the same conditions.

**Figure 6:**
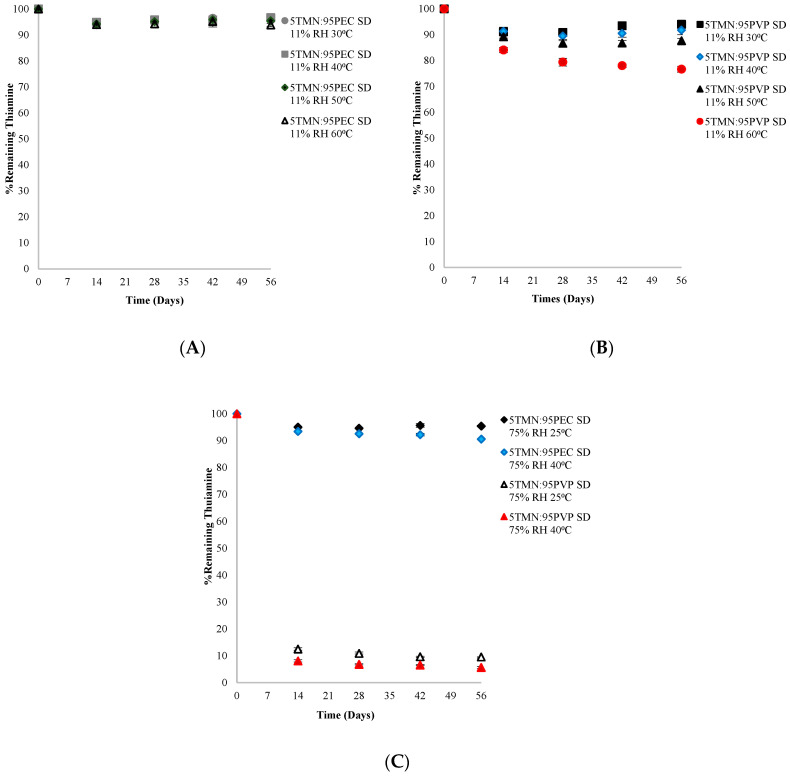
Chemical stability of thiamine (**A**) in 5TMN:95PEC solid dispersions (SD), (**B**) in 5TMN:95PVP dispersions (SD) stored at 11% RH and 30–60 °C for 56 days, and (**C**) in 5TMN:95PEC solid dispersions (SD) and 5TMN:95PVP dispersions stored at 75% RH and 25 or 40 °C for 56 days.

Thiamine degradation was also monitored in amorphous solid dispersions: in 5TMN:95PEC and 5TMN:95PVP solid dispersions at 11% RH and four different temperatures (30, 40, 50 and 60 °C), storage conditions in which no evidence of TMN crystallization was found for the duration of the study (Figure 2). Up to ~5% thiamine degradation occurred in the amorphous 5TMN:95PEC dispersions stored at 11% RH over the course of the 8-week storage study, with no significant differences in thiamine degradation found between storage temperatures (Figure 6A). Despite no significant difference in moisture sorption (amounting to <2% w/w) at these 11% RH storage conditions (as shown in Figure 5B), more thiamine degraded in the amorphous 5TMN:95PVP dispersions, with thiamine degradation increasing as temperature increased and RH was maintained at 11% RH (Figure 6B). Nearly 14% of thiamine degraded in the 5TMN:95PVP dispersions stored at 11% RH and 50 °C over the duration of the study, and 23% of thiamine degraded in the 5TMN:95PVP amorphous dispersions stored at 60 °C (Figure 6B). Thus, polymer type had a notable influence on the chemical stability of thiamine in the amorphous solid dispersions independent of moisture sorption, with the PEC polymer better stabilizing amorphous thiamine to chemical degradation than PVP at the 11% RH storage conditions.

Increasing the storage RH to 75% RH resulted in drastic differences in thiamine degradation between the 5TMN:95PEC and 5TMN:95PVP amorphous dispersions (Figure 6C), with thiamine being significantly (*p* < 0.05) more stable in the dispersions made with PEC. In the 75% RH environment, ~5% and ~9% of thiamine had degraded in the TMN:PEC dispersions stored at 25 °C and 40 °C, respectively, over the 8-week duration of the study. In contrast, after only 2 weeks of storage at 75% RH, 87% and 92% of thiamine had degraded, at 25 and 40 °C respectively, in the 5TMN:95PVP dispersions. After this 2-week period, thiamine continued to slowly degrade in these TMN:PVP dispersions, and only ~10% and ~6% of thiamine remained at the end of the 8-week study following storage at 75% RH and 25 or 40 °C, respectively (Figure 6C). The dramatic increase in thiamine degradation in the 5TMN:95PVP dispersions stored at the higher RH was attributed to the solubilization of TMN in the 95% PVP dispersions due to the hygroscopicity of PVP (Figure 5), similarly to the increased degradation of dissolved crystalline TMN in the physical mixtures with PVP stored at this higher RH (Figure 4B). However, even in the samples stored at 11% RH, where water content was much lower and not a differentiating factor, amorphous TMN was more chemically stable in the presence of PEC than PVP. This was presumably due to stronger/more extensive intermolecular interactions between TMN and PEC than between TMN and PVP (Figure 3), consistent with previous studies on the effect of intermolecular interactions on chemical stability of components within amorphous solid dispersions [10,17]. In general, thiamine was less chemically stable in the amorphous state than in the crystalline state, and the chemical stability of thiamine in amorphous solid dispersions was dependent on polymer type due to both intermolecular interactions with the polymer and the variable hygroscopicity of the systems in the different storage environments. To further explore the differences in chemical stability within the amorphous solid dispersions, a series of additional studies were completed.

### 2.4. Effect of Glass Transition Temperature on Thiamine Stability

Chemical stability of amorphous materials, including thiamine, is often related to T_g_: samples with lower T_g_ values often have lower chemical stabilities (enhanced reactivities) due to increased molecular mobility especially above the T_g_ [9,18,19]. Although the T_g_ of pure amorphous TMN could not be measured due to its inability to amorphize in the absence of a polymer, using the Boyer-Beaman rule, (T_g_ = 2/3 * T_m_) [20], the T_g_ of TMN was estimated as ~40 °C. The T_g_ values of PVP and PEC are reported to be 134 and 90 °C, respectively [8], indicating that PVP-containing lyophiles should have higher T_g_ values than PEC-containing lyophiles, all other factors remaining constant. Applying the Fox and Gordon–Taylor equations, which are used to predict the T_g_ values of systems containing more than one component as a function of weight fractions of the components [21,22], it can be assumed that as the proportion of polymer increased in the amorphous TMN:polymer dispersions, their T_g_ values also increased, due to the higher T_g_ values of the polymers compared to TMN. Accordingly, immediately following lyophilization, the T_g_ values of 5TMN:95PVP and 10TMN:90PVP dispersions were 65 and 60 °C, respectively (Table 1). Following storage of the 5TMN:95PVP dispersions at 11% RH and either 30 or 60 °C, their T_g_ values were measured as 49 and 52 °C, respectively (Table 1). Increasing the moisture contents of samples, such as by exposing them to humidity in the environment, decreases their T_g_ values (refer to the Fox and Gordon-Taylor equations: water has a T_g_ of −137 °C [19,21,22]). Storing the dry lyophiles of 5TMN:95PVP at 11% RH resulted in enough moisture sorption to decrease the T_g_ from the initial 65 °C to 49–52 °C. The TMN:PVP dispersions stored at 11% RH and 60 °C had absorbed enough water to depress their T_g_ values below the storage temperature, indicating that these dispersions were in the supercooled liquid state in this 60 °C storage condition. This change in state from the glassy amorphous state at lower temperatures to the supercooled liquid state at 60 °C corresponded to a significant increase in thiamine degradation in 5TMN:95PVP dispersions after 56 days of storage at 60 °C (24% thiamine degradation), compared to that in 5TMN:95PVP dispersions stored at lower temperatures (<14% thiamine degradation) (Figure 6B). These results are consistent with the concept that amorphous materials are more stable in the glassy state than in the supercooled liquid state due to differences in molecular mobility [18]. The T_g_s of the PEC-containing lyophiles were difficult to identify in the DSC scans, attributed to the polydispersity of the PEC.

**Figure 7:**
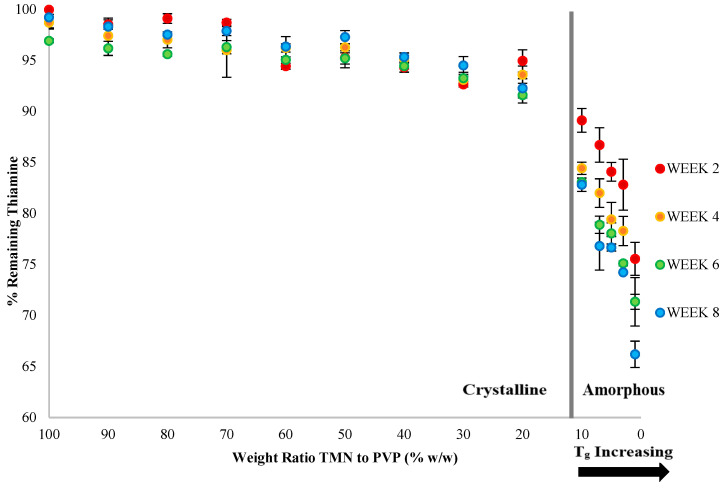
Chemical stability of thiamine in various ratios of TMN to PVP solid dispersions stored at 11% RH and 60 °C for 56 days.

Interestingly, thiamine was more chemically stable in the 10TMN:90PVP dispersions than in the 5TMN:95PVP dispersions (Figure 7) at storage temperatures below the T_g_, despite the lower T_g_ of the 10TMN:90PVP dispersion, indicating that factors beyond T_g_ are likely contributing to the stability differences of the vitamin in different amorphous dispersions especially in the glassy state. A number of studies have shown that T_g_ does not always correlate to chemical stability [10,23,24,25,26]. For example, aspartame chemical stability in the presence of PVP did not change even when T_g_ was increased [23], ascorbic acid exhibited degradation even in the glassy state [24], and thiamine degraded more in amorphous TClHCl:polymer dispersions with the highest T_g_ values than in these dispersions with lower T_g_ values [10]. Thus, although there was some correlation of T_g_ and chemical stability within the same formulation (5TMN:95PVP) in the current study, with the most degradation observed in the amorphous dispersion stored at a temperature above its T_g_, the T_g_ was not the only factor accounting for differences in chemical stability across different polymer (PVP vs. PEC) or vitamin:polymer ratio formulations.

### 2.5. Effect of Vitamin:Polymer Ratio in Solid Dispersions on Thiamine Stability

The chemical stability of amorphous thiamine has also been shown to relate to the polymer proportion in TClHCl:PVP solid dispersions [10]. To explore this possibility with TMN, PVP dispersions composed of increasing ratios of TMN (1–100%) were lyophilized and stored at 11% RH and 60 °C for 56 days. The samples were analyzed for physical state over the duration of the experiment, and at least 90% PVP was needed to amorphize TMN (Figure 2A). These initially amorphous TMN dispersions containing ≥90% PVP remained amorphous for the duration of the study at this storage condition. The percent thiamine remaining in these dispersions throughout the storage study was also documented to correlate the physical state of the vitamin with its chemical reactivity. The PVP dispersions containing ≤10% TMN remained amorphous and thus enabled documentation of thiamine stability in the amorphous state, whereas TMN crystallized in the PVP dispersions containing >10% TMN and thus the stability of thiamine in these dispersions would have been influenced by the crystalline state of the vitamin in the presence of PVP.

Significant (*p* < 0.05) differences in thiamine stability were found between the TMN:PVP dispersions in which TMN had crystallized and those that remained amorphous (Figure 7), with thiamine degrading significantly (*p* < 0.05) more in the amorphous systems. Of the TMN:PVP systems in which TMN had crystallized, significantly (*p* < 0.05) more thiamine had degraded in the 20TMN:80PVP dispersions by the end of the experiment (day 56, 8% degradation) than in the dispersions containing higher proportions of TMN. A similar observation was found in a previous study, in which only partial crystallization (some amorphous thiamine still present) led to some degradation in a crystallized dispersion [10]. 

Interestingly, more thiamine degradation was found in the physical mixtures of 5% crystalline TMN with 95% PVP at 60 °C and 11% RH (Figure 6B) than in the 5TMN:95PVPdispersions that had been co-lyophilized (in which the TMN had recrystallized) and stored in the same conditions (Figure 7), indicating that the sample preparation and resulting matrix also influenced vitamin stability. However, in the TMN:PVP dispersions prepared by lyophilization, a marked difference was found in the chemical stability of thiamine between systems that remained amorphous and those in which TMN had crystallized. In the TMN:PVP dispersions that remained amorphous (Figure 7), as the proportion of PVP in the dispersion increased, the thiamine stability decreased, consistent with some previous studies on chemical stability of vitamins (thiamine from TClHCl and ascorbic acid) as a function of proportion of PVP in amorphous dispersions [10,24]. Over the 8-week study, 23%, 23%, and 26% of thiamine degraded in the TMN:PVP dispersions containing 7%, 5%, and 3% TMN, respectively (Figure 7). The most thiamine degradation (34%) was found in the amorphous TMN:PVP dispersions containing only 1% TMN (Figure 7). This finding is important because thiamine fortification or enrichment levels in foods are low, and thiamine appears to be most reactive (and least stable) at low proportions in amorphous systems.

To further explore differences in thiamine stability in amorphous solid dispersions with increasing ratios of PVP, reaction kinetics of thiamine degradation were calculated. Thiamine is known to degrade in a first-order or pseudo-first order reaction [7,27]. Reaction rate constants (k_obs_) were calculated for the samples with different TMN and PVP ratios up to 20% TMN (higher TMN proportions did not degrade enough in the timeframe of the current study for accurate calculations). The k_obs_ values increased from 0.0038 to 0.0055 day^−1^ as PVP proportion increased from 90% to 99% (Table 2). However, as the PVP ratio decreased to 80%, in which TMN had at least partially crystallized, the k_obs_ value was only 0.0018 day^−1^. The t_90_ value (time where 90% of the initial concentration of thiamine remained) in this 20TMN:80PVP was 58.5 days, whereas the t_90_ values of dispersions in which TMN remained in the amorphous state were all less than 28 days (Table 2), indicating the relatively higher stability of the crystallized TMN compared to the amorphous samples.

It is important to note that the t_90_ value decreased as PVP proportion increased in the amorphous dispersions (i.e., 28 days and 19 days in 90% and 99% PVP dispersions, respectively) (Table 2). Since the observation that increasing amounts of PVP led to increased thiamine degradation could not be attributed to T_g_, this was instead explained by a kinetic model proposed by Waterman, et al. [28]. This model proposes that the degradation rate of a drug is affected by the drug to excipient ratio only if the excipient is in excess, wherein a greater surface area of contact between the drug and excipient leads to greater drug degradation [28]. This mechanism has been shown previously to occur in the case of thiamine in TClHCl:PVP dispersions [10] and is also presumably occurring in the current study. As the number of PVP molecules increased relative to TMN, thiamine–polymer interactions increased, leading to a decrease in thiamine–thiamine interactions. The thiamine–polymer interactions were destructive to the chemical stability of thiamine and led to the increased rate of thiamine degradation (Table 2). Thus, thiamine stability was dictated more by molecular interactions between TMN and polymer than by T_g_ in the glassy state.

### 2.6. Comparison of Thiamine Stability in TMN and TClHCl Solid Dispersions

#### 2.6.1. Physical Stability

TMN and TClHCl are both used commercially in the food and pharmaceutical industries; therefore, comparing the physical and chemical stability of these two salt forms of thiamine in the amorphous state is advantageous for many food and pharmaceutical products. When using PEC and PVP to amorphize thiamine, TMN required more polymer than TClHCl for amorphization regardless of the polymer type used: TClHCl was successfully amorphized with a minimum of 40% and 60% of PEC and PVP, respectively [8], while at least 80% PEC and 90% PVP were required to amorphize TMN (Figure 1). Polymers contribute to the amorphization of crystalline materials during lyophilization by disrupting thiamine–thiamine interactions in the crystal lattice structure and instead forming thiamine–polymer interactions. However, the nature of the counterion present in the crystal lattice (i.e., nitrate in TMN vs. chloride in TClHCl) plays a role in the strength of interactions and packing of the crystal lattice [29]; thus the differences in amorphization tendency, physical stability, and other physicochemical properties (Table 3) between the two salt forms of thiamine are likely to be related to the differences in counterions. The presence of the additional HCl in TClHCl could have led to stronger and/or more extensive hydrogen bonding between TClHCl and the polymers compared to TMN, as evident in the larger hydroxyl region peak shifts in the FTIR spectra of the TClHCl:PEC dispersions [8] than the TMN:PEC dispersions (Figure 3A). The additional HCl in TClHCl also contributes to the much higher solubility of TClHCl compared to TMN (Table 3). This enhanced intermolecular interaction network between TClHCl and PEC compared to TMN and PEC likely contributed to the higher proportions of polymer necessary to amorphize TMN than TClHCl. Additionally, the T_g_ values of TMN and TClHCl are 40 and 74 °C, respectively, estimated using the Boyer–Beaman rule (Table 3); thus, more polymer was necessary to increase the T_g_ of the TMN solid dispersions to the same extent as in the TClHCl solid dispersions. The difference in T_g_ between the two salt forms may have also contributed to the higher proportions of polymer necessary to amorphize TMN than TClHCl.

When comparing the physical stability of amorphous thiamine in solid dispersions with 95% polymer, PEC and PVP prevented crystallization of both TMN and TClHCl in the dispersions over the 8-week duration of the study when stored at 11% RH and 30 °C–60 °C. In both TMN and TClHCl dispersions, less PEC was required to amorphize the vitamin and also better stabilized amorphous thiamine than PVP, indicating an advantage for interactions between thiamine and PEC compared to thiamine and PVP. The increased physical stability of thiamine in the presence of PEC also correlated to increased chemical stability when compared to PVP dispersions. Differences in physical stability of amorphous thiamine were related to the salt form of thiamine (counterion present), type of polymer, and amount of polymer present in the dispersion, mainly due to differences in the extent of hydrogen bonding between thiamine and the polymer.

#### 2.6.2. Chemical Stability

Thiamine was more chemically stable in the crystalline state than in the amorphous state for both TMN and TClHCl physically blended or co-lyophilized with PEC or PVP. Increasing the storage RH to 75% RH, compared to 11% RH, increased the degradation of thiamine in physical mixtures of TMN or TClHCl blended with PVP, attributed to partial dissolution of the vitamin in the plasticized PVP matrix at this RH. Significantly more (*p* < 0.05) thiamine degraded in TMN blends than in TClHCl blends with PVP after 8 weeks of storage at 75% RH and 40 °C (27% and 76% thiamine remaining, respectively). A contributing factor to the difference in thiamine loss between TMN and TClHCl in these systems could be attributed to differences in the pre-lyophilization pH of the samples, which were 5.2 and 3.8 in 5TMN:95PVP and 5TClHCl:95PVP solutions, respectively (in 10 mL of H_2_O at 25 °C). Thiamine is known to be much more stable in acidic conditions [2]. Although pre-lyophilization pH does not always correlate to pH of the dispersion [32], the role of pH is still worth considering. Voelker, Miller, Running, Taylor and Mauer [5] investigated TMN and TClHCl degradation in solutions, and due to the pH of solutions as a function of thiamine salt form resulting from differences in counterion in solution (similar to the pre-lyophilization pHs in the current study), thiamine was much more stable in solutions of TClHCl than TMN. No significant thiamine degradation occurred in TClHCl solutions stored at 40 °C for 174 days, but only 44–87% of thiamine (dependent on starting concentration) remained in TMN solutions after 63 days of storage at 40 °C [5]. 

**Figure 8:**
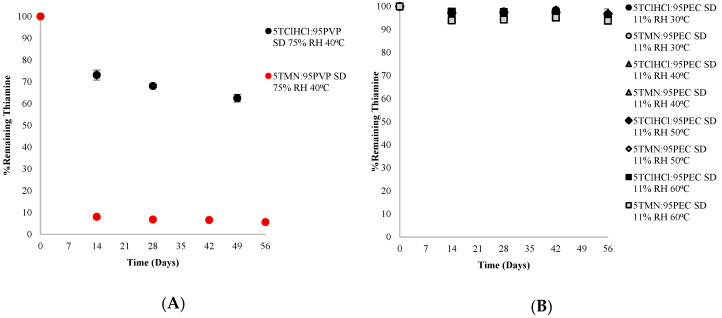

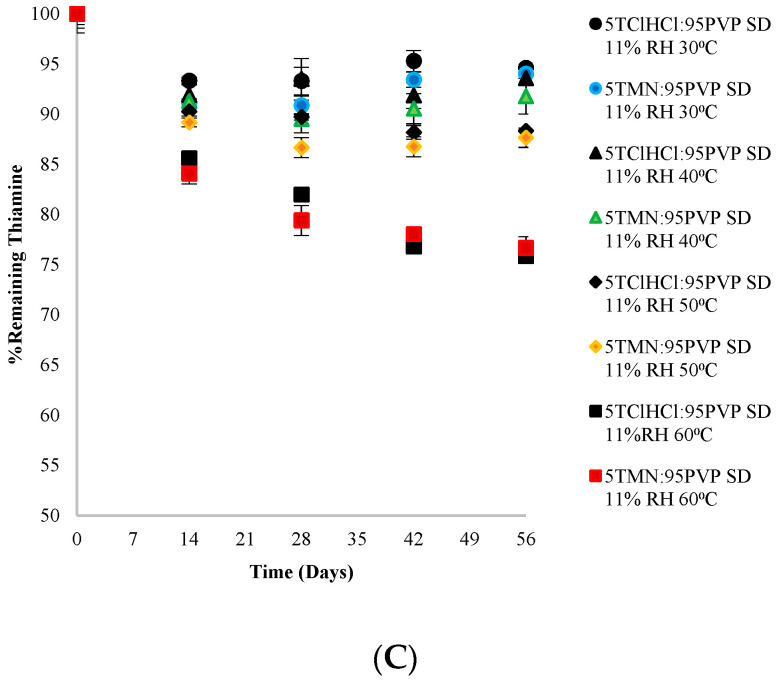
Comparison of chemical stability of TMN and TClHCl amorphous solid dispersions (SD) with 95% polymer: (**A**) SDs with 95% PVP and stored at 75% RH and 40 °C; (**B**) SDs with 95% PEC and stored at 11% RH and 30–60 °C; and (**C**) SDs with 95% PVP and stored at 11% RH and 30–60 °C.

This pH-related trend in thiamine stability was also found in the amorphous solid dispersions of 5TMN:95PVP and 5TClHCl:95PVP stored at 75% RH and 40 °C, wherein significantly more thiamine degraded in 5TMN:95PVP dispersions than in 5TClHCl:95PVP dispersions (7% and 68% thiamine remaining after 28 days of storage, respectively) (Figure 8A). As in the crystalline physical mixtures, the hygroscopic nature of PVP led to considerable moisture sorption in which hydration of both TMN and TClHCl occurred, and the differences in thiamine degradation between TMN and TClHCl in the amorphous solid dispersions were attributed to pH differences: less thiamine degraded in the more acidic systems containing TClHCl than in those containing TMN. The degradation pathway of thiamine differs as a function of pH, and thus the degradation products formed in each condition also differ [5]. Differences in degradation peaks on HPLC chromatograms of degraded 5TMN:95PVP and 5TClHCl:95PVP samples indicate that degradation pathway may have differed between the two samples, which suggests that pH was potential factor for differences in stability between TMN and TClHCl in storage conditions that resulted in dissolved thiamine in the samples.

However, thiamine degradation did not significantly differ between the comparable formulations of TMN and TClHCl dispersions with PEC or PVP stored at 11% RH, where less vitamin hydration would have occurred (Figure 8B,C). When chemical stability of thiamine in TMN and TClHCl dispersions prepared with increasing ratios of PVP were compared in samples stored at 11% RH, thiamine stability and reaction kinetics in comparable formulations and storage conditions were fairly similar and most often not significantly different regardless of thiamine salt form. For example, in dispersions containing 99% PVP stored for 56 days at 11% RH and 60 °C, 66% and 65% thiamine remained and k_obs_ = 0.0055 and 0.0062 day^−1^ in TMN and TClHCl dispersions, respectively (Table 2). In general, both salt forms of thiamine had similar degradation behavior in the amorphous glassy solid state, and polymer type had the same impact on thiamine stability regardless of thiamine salt form.

## 3. Materials and Methods

### 3.1. Materials

Crystalline thiamine mononitrate (TMN) was purchased from Spectrum Chemical Mfg. Corp. (New Brunswick, NJ, USA). The polymers used in the study were pectin (PEC) from citrus peel with a ~61% degree of esterification and polyvinylpyrrolidone (PVP) with a molecular weight of 40,000, both of which were purchased from Sigma-Aldrich Inc. (St. Louis, MO, USA). The RH conditions (reported here at 25 °C) were created in desiccators using saturated salt solutions of lithium chloride (LiCl, 11% RH) (EMD Millipore, Billerica, MA, USA) or sodium chloride (NaCl, 75% RH) (Sigma-Aldrich Inc.). HPLC grade trifluoroacetic acid (TFA) was purchased from Sigma-Aldrich Inc., and acetonitrile was purchased from Fisher Scientific Co., LLC (Pittsburgh, PA, USA). A Barnstead E-Pure ultrapure water purification system (ThermoScientific, Waltham, MA, USA) with a resistivity of ~17.5 MΩ·cm was used to deionize and purify all water used in this study.

### 3.2. Preparations of TMN Solid Dispersions via Lyophilization

TMN:polymer dispersions were prepared by lyophilization using our previously reported method [8,10]. First, 100 mg total solid content was dissolved in 10 mL of deionized water (w/v). To investigate the effect of vitamin proportion on physical and chemical stability, 14 different mass ratios of TMN to polymer (both PEC and PVP) (1%, 3%, 5%, 7%, 10%, 20%, 30%, 40%, 50%, 60%, 70%, 80%, 90%, and 100% TMN) were prepared by varying the TMN:polymer ratios while maintaining the total solids content at 100 mg. All the samples were prepared in triplicate. TMN:polymer solutions were mixed with a Roto-Shake Genie^®^ SI-1100 (Scientific Industries, Inc., Bohemia, NY, USA) for 10 min. The pHs of the solutions were measured with an Orion Star A211 pH meter (ThermoScientific Inc.). The solutions were then frozen overnight at −20 °C prior to lyophilization. Frozen samples were then loaded into a VirTis Genesis 25ES shelf freeze dryer (SP Scientific, Stone Ridge, NY, USA). Initially, samples were frozen for 6 h at −40 °C and 300 mTorr. Then, the first drying step was initiated in order to remove the bulk of water via sublimation at −40 °C by maintaining the vacuum at 150 mTorr for 24 h (primary drying). In the secondary drying step, samples were held for 9 h at each step in 10 °C intervals from −40 to 20 °C. Finally, the samples were held at 25 °C and 300 mTorr for 6 h. Immediately after the freeze-drying cycle was complete, samples were placed into the RH-controlled desiccators. 

### 3.3. Storage Treatments

Samples were stored in desiccators at select temperature and RH conditions. Four different temperatures at the same RH were chosen for the purpose of calculating reaction kinetics of thiamine degradation: 11% RH and 30, 40, 50 and 60 °C. In addition to these conditions, a higher storage RH (75%) at two different temperatures (25 and 40 °C) was selected to investigate the effect of high RH on thiamine degradation. Saturated salt solutions were used to control the RH in desiccators, and RH values were verified by an AquaLab 4TE water activity meter (Decagon Devices Inc., Pullman, WA, USA). Temperature conditions were controlled by using water jacketed incubators (Forma Scientific, Inc., Marietta, OH, USA) in which the desiccators were placed. Samples were stored for up to eight weeks, with a subset removed biweekly for HPLC analysis. This subset was then discarded after analysis.

### 3.4. Powder X-ray Diffraction (PXRD)

A Rigaku Smartlab^TM^ diffractometer (Rigaku Americas, TX, USA) equipped with a Cu-Kα radiation source and D/tex ultra-detector was used to analyze the physical structure of the starting ingredients and initial solid dispersions. Additionally, TMN:polymer solid dispersions stored at select conditions were analyzed over time. Scans were generated between 5 and 40° 2θ with an increment of 0.02° and a rate of 4° per min. Structural distinction between amorphous and crystalline TMN was determined based on the PXRD patterns. Samples that exhibited a diffuse halo were considered to be PXRD amorphous, while samples in which sharp peaks greater than two standard deviations above the baseline were observed on the diffractogram were labeled as crystalline.

### 3.5. Fourier Transform Infrared Spectroscopy (FTIR)

Samples were analyzed by FTIR to determine the presence and extent of hydrogen bonding and intermolecular interactions between thiamine and the polymer in the solid dispersions. A Nexus 670 FTIR spectrometer (ThermoNicolet, Madison, WI, USA) equipped with an MCTA detector and a DRIFTS Avatar Diffuse Reflectance Smart Accessory (ThermoElectron Corp., Madison, WI, USA) was used to collect FTIR spectra of samples, as previously described by Arioglu-Tuncil, Bhardwaj, Taylor and Mauer [8]. The spectra of samples were analyzed using OMNIC software (ThermoElectron Corp.).

### 3.6. Chemical Stability Determination with HPLC

A Waters 2690SM (Waters Corp., Milford, MA, USA) HPLC with a Waters Xselect HSS T3 (3.5 µm, 4.6 × 100 mm) column and a Waters 2996 photodiode array detector were used for thiamine quantification in samples containing crystalline TMN, physical blends of crystalline TMN with polymers, and thiamine:polymer solid dispersions. The amount of thiamine remaining was quantified by the standard curve prepared at a concentration range 0.005–1 mg/mL prior to analysis (r^2^ = 0.9997–1.0000). Samples were diluted with solvent to a final thiamine concentration of 0.25–0.5 mg/mL and filtered through a 0.2 µm syringe filter. Acetonitrile (solvent A) and water with 0.1% TFA (solvent B) were used as mobile phases. The following gradient method was adapted from Xia, et al. [33] and performed as follows: 0/100 at 0 min (immediate), 3/97 at 4 min (linear), 10/90 at 6 min (linear), 0/100 10 min (linear), and 0/100 from 10 to 15 min (immediate). The flow rate and the injection volume used were 1 mL/min and 10 μL, respectively. Samples were scanned between 235–400 nm, and the integration was conducted at 254 nm using Masslynx V4.1 software (Waters Corp.). 

### 3.7. Moisture Sorption Isotherm Analysis

A SPSx-1 μ Dynamic Vapor Sorption Analyzer (Project Messtechnik, Ulm, Germany) was used to obtain moisture sorption profiles of individual ingredients and solid dispersions with 95% polymer. Samples (100–300 mg) were loaded into aluminum pans in a 24-ring sample holder. Equilibrium criteria and maximum step time were set at a weight change of 0.001% in 30 min and 12 h, respectively. Samples were first equilibrated at 0% RH for 12 h and then analyzed from 5% to 95% RH at 25 °C with a 5% RH step size. All samples were measured in duplicate.

### 3.8. Determination of Glass Transition Temperature by Differential Scanning Calorimetry (DSC)

Thermal analyses of the samples were conducted using a Discovery DSC equipped with a refrigerated cooling accessory (TA Instruments, New Castle, DE, USA). Nitrogen served as the purge gas at a rate of 50 mL/min. Approximately 7–12 mg of sample was weighed and hermetically sealed into Tzero pans (TA Instruments). Samples were heated from −20 °C to a temperature 20–30 °C higher than the expected T_g_ values at a rate of 20 °C/min, followed by cooling to −20 °C at a rate of 10 °C/min. Then, the second heating scan was applied to 150 °C at 20 °C/min. T_g_ was determined in this second heating step (where a baseline shift occurred in the endothermic direction) using TRIOS software. 

### 3.9. Reaction Kinetics Calculations

Thiamine has been observed to follow first order or pseudo-first order reaction kinetics [5,7,27,34]. Experimental data were fitted to the following equation to calculate reaction rate constants:

(1) $$\ln\frac{x\;}{x_{0}}=-kt$$

where *x* corresponds to thiamine concentration at time *t* (days), *x*_0_ is the initial thiamine concentration, and *k* is the reaction rate constant (days^−1^). The value of t_90_, the time at which 90% of the initial concentration of thiamine remains, was evaluated using the following equation:

(2) $$t_{90}=\frac{\ln\left(0.9\right)}{-k}$$

where *k* is the reaction rate constant (days^−1^).

### 3.10. Statistical Analysis

All analyses were performed in triplicate, unless otherwise noted, and data are presented as mean ± standard deviation. SAS Software Version 9.4 (SAS Institute, Cary, NC, USA) was used to conduct statistical analyses. Analysis of variance (ANOVA) was performed at α = 0.05 significance level to determine differences among the samples and controls. Tukey’s multiple comparison test (α = 0.05) was used to test whether the samples were statistically different. 

## 4. Conclusions

Amorphization of TMN was achieved when at least 80% of PEC or 90% of PVP were used in the dispersion formulations, and both polymers were effective at inhibiting TMN crystallization in the solid dispersions stored at 11% RH (30–60 °C) for the entirety of the 8-week study. At a higher storage RH (75%RH), PEC inhibited the crystallization of TMN in the initially amorphous solid dispersions, but the higher moisture sorption in the PVP dispersions led to hydration of the TMN. While no significant thiamine loss was found in crystalline TMN, thiamine degradation was accelerated when thiamine was in the amorphous form, and further accelerated when the TMN dissolved in the polymer matrix. Thiamine was less labile in PEC dispersions than in PVP dispersions, attributed to the greater stabilizing effects of noncovalent intermolecular interactions between the PEC and TMN and the more acidic nature of the PEC polymer. Comparing the physical and chemical stability of thiamine from the two most common ingredient salt forms of the vitamin used in food and pharmaceutical ingredients (TMN and TClHCl) led to the following understanding of counterion and polymer co-formulation effects on thiamine stability in dispersions:

- Thiamine was less stable when present at low proportions in a solid dispersion than when present at higher proportions (a concerning factor when delivering low concentrations of thiamine in solid formulations, and an indicator that the sample T_g_ is not the main factor in governing thiamine stability in amorphous systems since the thiamine was most labile in the dispersions with the highest T_gs_);
- Thiamine was most stable in the crystalline form, less stable in the amorphous form, and degraded most rapidly when present in (concentrated) solutions (such as in the physical mixtures and dispersions of TMN with PVP that were stored at 75% RH and 40 °C);
- Despite the higher solubility of TClHCl compared to TMN, thiamine was more stable in the amorphous vitamin:polymer dispersions made with TClHCl than with TMN. The enhanced stability of thiamine in the TClHCl:polymer dispersions was attributed to not only the increased noncovalent intermolecular interactions between the TClHCl and polymer compared to the TMN-polymer interactions (TClHCl has an additional hydrogen bond donor), but also to the more acidic pHs created by the HCl counterion than the nitrate counterion.

TMN is the more common ingredient form of thiamine used to fortify or enrich solid products. While thiamine degrades via different pH-dependent pathways, which should be explored when considering formulations for delivering thiamine in foods and dietary supplements given not only the need to deliver the amount of vitamin claimed on the label but also the possible sensory-related impacts of thiamine degradation, the finding that thiamine was more stable in TClHCl:polymer solid dispersions than in TMN:polymer solid dispersions supports the consideration of using TClHCl instead of TMN as the form of thiamine in products wherein the vitamin is present in the final formulation in amorphous form.

## Figures and Tables

**Table 1:** Onset glass transition temperatures of TMN:PVP solid dispersions after lyophilization and equilibration at 11% RH and 30 or 60 °C. Superscript letters denote statistical significance.

Sample	Storage Condition	Onset T_g_ (°C)
5TMN:95PVP	‘As is’ following lyophilization	65 ± 7 ^B^
5TMN:95PVP	11% RH and 30 °C	49 ± 1 ^A^
5TMN:95PVP	11% RH and 60 °C	52 ± 3 ^A^
10TMN:90PVP	‘As is’ following lyophilization	60 ± 4 ^B^

**Table 2:** Reaction rate constants and t_90_ values for thiamine degradation in TMN and TClHCl solid dispersions prepared with different proportions of PVP when stored at 11% RH and 60 °C.

Thiamine Salt Form	% PVP	k_obs_ (day^−1^)	R^2^	t_90_ * (days)
**TMN**	99	0.0055	0.7981	19.2
	97	0.0054	0.9217	19.5
	95	0.0050	0.8637	21.1
	93	0.0049	0.9371	21.5
	90	0.0038	0.9155	27.7
	80	0.0018	0.9464	58.5
**TClHCl ^1^**	99	0.0062	0.96	17
	97	0.0047	0.95	22
	95	0.0038	0.95	28
	93	0.0032	0.93	33
	90	0.0025	0.95	42
	80	0.0015	0.92	70

^1^ Arioglu-Tuncil, Voelker, Taylor and Mauer [10]. * t_90_: Time when 90% of the initial concentration of thiamine remained.

**Table 3:** Solid state property comparison between TMN and TClHCl.

	Thiamine Mononitrate	Thiamine Chloride Hydrochloride
Structure	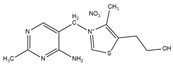	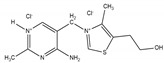
Molecular weight ^1^	327.36 g/mol	337.26 g/mol
Melting point ^1^	196–200 °C	248 °C
T_g_^2^	40 °C	74 °C
Deliquescence point (RH_0_) ^3^	98.5% RH	88% RH
Aqueous solubility ^4^	30 mg/mL	570 mg/mL

^1^ ChemSpider [30]. ^2^ Calculated using Boyer–Beaman rule (T_g_ = 2/3 × T_m_) [20]. ^3^ Hiatt, et al. [31]. ^4^ Voelker, Miller, Running, Taylor and Mauer [5].

## Abbreviations

DSC	Differential scanning calorimetry
FTIR	Fourier Transform infrared spectroscopy
HBA	Hydrogen bond acceptor
HBD	Hydrogen bond donor
HPLC	High-performance liquid chromatography
PEC	Pectin
PM	Physical mixture
PVP	Poly(vinylpyrrolidone)
PXRD	Powder X-ray diffraction
RH	Relative humidity
SD	Solid dispersion
TClHCl	Thiamine chloride hydrochloride
TMN	Thiamine mononitrate
TFA	Trifluoroacetic acid
T_g_	Glass transition temperature
